# Supplementary material for: Genome-Wide Analysis of Nubian Ibex Reveals Candidate Positively Selected Genes That Contribute to Its Adaptation to the Desert Environment
Source: Animals (Basel). 2020 Nov 22;10(11):2181. doi: 10.3390/ani10112181 (PMC7700370; doi:10.3390/ani10112181)
Supplement: Supplementary file 1 [file animals-10-02181-s001.zip › Supplemental File S1. Additional information for Nubian ibexes.docx]

**Information on *C. nubiana* individuals used in this study**

DNA samples for 21 *C. nubiana* individuals were obtained from the National Zoological Garden, Pretoria, South Africa. The animals were originally imported from Northern Africa and were kept in a zoo until they died of natural causes. Genetic diversity analysis of the DNA samples using mitochondrial DNA (mtDNA) D-loop in our lab (unpublished) showed that the *C. nubiana* individuals were very similar hence sample from one individual was selected to act as a representative. The data from the two additional *C. nubiana* individuals were downloaded from the National Center for Biotechnology Information database (https://www.ncbi.nlm.nih.gov/) under Sequence Read Archive (SRA) accession number [SRR8437789](https://trace.ncbi.nlm.nih.gov/Traces/sra/?run=SRR8437789) and [SRR8437792](https://trace.ncbi.nlm.nih.gov/Traces/sra/?run=SRR8437792). One *C. nubiana* individual (SRR8437789) was obtained from Sinai in Egypt, while the other (SRR8437792) was obtained from Saudi Arabia[1].

**References**

1. Grossen, C.; Guillaume, F.; Keller, L.F.; Croll, D. Purging of highly deleterious mutations through severe bottlenecks in Alpine ibex. *Nature Communications* **2020**, *11*, 1001, doi:10.1038/s41467-020-14803-1.
