# Supplementary material for: Genome-Wide Analysis of Nubian Ibex Reveals Candidate Positively Selected Genes That Contribute to Its Adaptation to the Desert Environment
Source: Animals (Basel). 2020 Nov 22;10(11):2181. doi: 10.3390/ani10112181 (PMC7700370; doi:10.3390/ani10112181)
Supplement: Supplementary file 1 [file animals-10-02181-s001.zip › Supplemental File S2. Data sources of the taxa used for positive selection analysis.docx]

Data sources of the taxa used for positive selection analysis

| Species name | Data source |
| --- | --- |
| *Bos taurus* (Cow) | ftp://ftp.ensembl.org/pub/release-97/fasta/bos_taurus/ |
| *Ovis aries* (Sheep) | ftp://ftp.ensembl.org/pub/release-97/fasta/ovis_aries/ |
| *Equus caballus* (Horse) | ftp://ftp.ensembl.org/pub/release-97/fasta/equus_caballus/ |
| *Equus asinus asinus* (Donkey) | ftp://ftp.ensembl.org/pub/release-97/fasta/equus_asinus_asinus/ |
| *Sus scrofa* (Pig) | ftp://ftp.ensembl.org/pub/release-97/fasta/sus_scrofa/ |
| *Pantera tigris altaica* (Tiger) | ftp://ftp.ensembl.org/pub/release-97/fasta/panthera_tigris_altaica/ |
| *Felis catus* (Cat) | ftp://ftp.ensembl.org/pub/release-97/fasta/felis_catus/ |
| *Canis familiaris* (Dog) | ftp://ftp.ensembl.org/pub/release-97/fasta/canis_familiaris/ |
| *Capra hircus* (Domestic goat) | ftp://ftp.ensembl.org/pub/release-97/fasta/capra_hircus/ |
| *Bos mutus* (Wild Yak) | ftp://ftp.ensembl.org/pub/release-97/fasta/bos_mutus/ |
| *Bison bison bison* (American Bison) | ftp://ftp.ensembl.org/pub/release-97/fasta/bison_bison_bison/ |
| *Ailuropoda melanoleuca*  (Krishnan & Panda) | ftp://ftp.ensembl.org/pub/release-97/fasta/ailuropoda_melanoleuca/ |
| *Bubalus bubalis* (Water Buffallo) | <ftp://ftp.ncbi.nlm.nih.gov/genomes/all/GCF/003/121/395/GCF_003121395.1_UOA_WB_1/> |
| *Pantholops hodgsonii* (Tibetian Antelope) | <ftp://ftp.ncbi.nlm.nih.gov/genomes/all/GCF/000/400/835/GCF_000400835.1_PHO1.0/> |
| *Capra aegagrus* (Bezoar) | <ftp://ftp.ncbi.nlm.nih.gov/genomes/all/GCA/000/978/405/GCA_000978405.1_CapAeg_1.0/> |
| *Capra nubiana* (Nubian ibex) | Generated and described in chapter 3 |
