# Supplementary material for: Genome-Wide Analysis of Nubian Ibex Reveals Candidate Positively Selected Genes That Contribute to Its Adaptation to the Desert Environment
Source: Animals (Basel). 2020 Nov 22;10(11):2181. doi: 10.3390/ani10112181 (PMC7700370; doi:10.3390/ani10112181)
Supplement: Supplementary file 1 [file animals-10-02181-s001.zip › Supplemental File S3. Codeml control file.docx]

**Codeml control file, Alternate model (H1)**

| **Input data and parameters** | **Description of parameters** | |
| --- | --- | --- |
| seqfile = Ibex-aligments.phy | **sequence data file name | |
| treefile =Ibex-trees.nwk | **result file name | |
| outfile =Codeml-output.txt | **tree structure file name | |
|  |  | |
| CodonFreq = 2 | ** 0:1/61 each, 1:F1X4, 2:F3X4, 3:codon table | |
| cleandata = 1 | ** remove sites with ambiguity data (1:yes, 0:no)? | |
| NSsites = 2 | ** 0:one w; 1:NearlyNeutral; 2:PositiveSelection; 3:discrete; | |
|  | ** 4:freqs; 5:gamma;6:2gamma;7:beta;8:beta&w;9:beta&gamma;10:3normal | |
| model = 2 | ** models for codons: | |
|  | * 0:one, 1:b, 2:2 or more dN/dS ratios for branches | |
|  |  | |
| fix omega = 0 | ** 1: omega or omega 1 fixed, 0: estimate | |
| omega = 1 | ** initial or fixed omega, for codons or codon-based AAs | |
| fix kappa = 0 | ** 1: kappa fixed, 0: kappa to be estimated | |
| kappa = 2 | ** initial or fixed kappa | |
| fix alpha = 1 | ** 0: estimate gamma shape parameter; 1: fix it at alpha | |
| alpha = 0 | ** initial or fixed alpha, 0:infinity (constant rate) | |
| clock = 0 | ** 0: no clock, unrooted tree, 1: clock, rooted tree | |
| runmode = 0 | **0: user tree; 1: semi-automatic; 2: automatic | |
|  | * 3: StepwiseAddition; (4,5):PerturbationNNI; -2: pairwise | |
|  |  | |
| Small Diff = .45e-6 | ** Default value. | |
| method = 1 | ** 0: simultaneous; 1: one branch at a time | |
| aaDist = 0 | ** 0:equal, +:geometric; -:linear, 1-6:G1974,Miyata,c,p,v,a, 7:AAClasses | |
| RateAncestor = 1 | ** (0,1,2): rates (alpha>0) or ancestral states (1 or 2) | |
| icode = 0 | ** 0:standard genetic code; 1:mammalian mt; 2-10:see below | |
| seqtype = 1 | ** 1:codons; 2:AAs; 3:codons-->AAs | |
| getSE = 0 | ** 0: don't want them, 1: want S.E.s of estimates | |
| noisy = 0 | ** 0,1,2,3,9: how much rubbish on the screen | |
| ndata = 1 | ** specifies the number of separate data sets in the file | |
| verbose = 1 | ** 1: detailed output, 0: concise output | |
| fix blength =0 | ** 0: ignore, -1: random, 1: initial, 2: fixed | |
|  | |  |

**Codeml control file, Null model (H0)**

| **Input data and parameters** | **Description of parameters** |
| --- | --- |
| seqfile = Ibex-aligments.phy | **sequence data file name |
| treefile =Ibex-trees.nwk | **result file name |
| outfile =Codeml-output.txt | **tree structure file name |
|  |  |
| CodonFreq = 2 | ** 0:1/61 each, 1:F1X4, 2:F3X4, 3:codon table |
| cleandata = 1 | ** remove sites with ambiguity data (1:yes, 0:no)? |
| NSsites = 2 | ** 0:one w; 1:NearlyNeutral; 2:PositiveSelection; 3:discrete; |
|  | ** 4:freqs; 5:gamma;6:2gamma;7:beta;8:beta&w;9:beta&gamma;10:3normal |
| model = 2 | ** models for codons: |
|  | * 0:one, 1:b, 2:2 or more dN/dS ratios for branches |
|  |  |
| fix omega = 1 | ** 1: omega or omega 1 fixed, 0: estimate |
| omega = 1 | ** initial or fixed omega, for codons or codon-based AAs |
| fix kappa = 0 | ** 1: kappa fixed, 0: kappa to be estimated |
| kappa = 2 | ** initial or fixed kappa |
| fix alpha = 1 | ** 0: estimate gamma shape parameter; 1: fix it at alpha |
| alpha = 0 | ** initial or fixed alpha, 0:infinity (constant rate) |
| clock = 0 | ** 0: no clock, unrooted tree, 1: clock, rooted tree |
| runmode = 0 | **0: user tree; 1: semi-automatic; 2: automatic |
|  | * 3: StepwiseAddition; (4,5):PerturbationNNI; -2: pairwise |
|  |  |
| Small Diff = .45e-6 | ** Default value. |
| method = 1 | ** 0: simultaneous; 1: one branch at a time |
| aaDist = 0 | ** 0:equal, +:geometric; -:linear, 1-6:G1974,Miyata,c,p,v,a, 7:AAClasses |
| RateAncestor = 1 | ** (0,1,2): rates (alpha>0) or ancestral states (1 or 2) |
| icode = 0 | ** 0:standard genetic code; 1:mammalian mt; 2-10:see below |
| seqtype = 1 | ** 1:codons; 2:AAs; 3:codons-->AAs |
| getSE = 0 | ** 0: don't want them, 1: want S.E.s of estimates |
| noisy = 0 | ** 0,1,2,3,9: how much rubbish on the screen |
| ndata = 1 | ** specifies the number of separate data sets in the file |
| verbose = 1 | ** 1: detailed output, 0: concise output |
| fix blength =0 | ** 0: ignore, -1: random, 1: initial, 2: fixed |
