# Supplementary material for: Genome-Wide Analysis of Nubian Ibex Reveals Candidate Positively Selected Genes That Contribute to Its Adaptation to the Desert Environment
Source: Animals (Basel). 2020 Nov 22;10(11):2181. doi: 10.3390/ani10112181 (PMC7700370; doi:10.3390/ani10112181)
Supplement: Supplementary file 1 [file animals-10-02181-s001.zip › Supplemental File S4. Positively selected amino acid sites and their functional impact.docx]

| **Amino acid under positive selection and the functional impact of the non-synonymous mutation** | | | | | |
| --- | --- | --- | --- | --- | --- |
|  | | **Bayes empirical Bayes analysis** | | **Polyphen-2 analysis** | |
| **Ensemble gene id** | **Gene name** | **Amino acid changes** | **Posterior probabilities** | **Score** | **Impact** |
| ENSCHIT00000003090 | Storkhead box 2 | T734V | 0.893 | 0.049 | benign |
|  |  | N835T | 0.814 | 0.065 | benign |
| ENSCHIT00000004084 | ATPase H+ transporting V1 subunit E2 | M72N | 0.998** | 0.711 | Possibly damaging |
| ENSCHIT00000004434 | olfactory receptor 2G2-like | F73T | 0.997** | 0.996 | probably damaging |
| ENSCHIT00000008957 | Serine protease 56 | Q424L | 0.917 | 0.001 | benign |
|  |  | R425G | 0.846 | 0.992 | probably damaging |
|  |  | R436W | 0.845 | 0.002 | benign |
|  |  | A548G | 0.739 | 0 | benign |
| ENSCHIT00000010253 | Matrix AAA peptidase interacting protein 1 | T76A | 0.835 | 0 | benign |
|  |  | Q93P | 0.943 | 0 | benign |
| ENSCHIT00000012782 | Putative olfactory receptor 52P1 | M67L | 0.924 | 0.889 | Possibly damaging |
| ENSCHIT00000015750 | Prostaglandin I2 synthase | A79M | 0.747 | 0.832 | possibly damaging |
|  |  | R320H | 0.831 | 0.816 | possibly damaging |
|  |  | D411E | 0.898 | 0 | benign |
| ENSCHIT00000018881 | F-box protein 21 | S603A | 0.864 | 0 | benign |
|  |  | E606G | 0.972* | 0.002 | benign |
|  |  | K615E | 0.974* | 0.11 | benign |
|  |  | K616R | 0.971* | 0.884 | possibly damaging |
|  |  | E620G | 0.999** | 0 | benign |
| ENSCHIT00000026283 | Zinc finger and SCAN domain containing 23 | P213N | 0.969* | 0.997 | probably damaging |
| ENSCHIT00000028977 | UV stimulated scaffold protein A | D361G | 0.917 | 0.992 | probably damaging |
|  |  | A517T | 0.897 | 0.001 | benign |
| ENSCHIT00000029782 | Leucine rich repeats and WD repeat domain containing 1 | T61M | 0.767 | 0.172 | benign |
|  |  | E99Q | 0.789 | 1 | probably damaging |
|  |  | A588T | 0.782 | 0.003 | benign |
| ENSCHIT00000030384 | F-box and WD repeat domain containing 2 | L82C | 0.954* | 0.998 | probably damaging |
| ENSCHIT00000000612 | Multimerin 2 | S214H | 0.821 | 0.997 | probably damaging |
|  |  | A559T | 0.541 | 0.009 | benign |
| ENSCHIT00000015914 | Toll like receptor adaptor molecule 2 | R43H | 0.56 | 0 | benign |
|  |  | I213N | 0.965* | 0.297 | benign |
| ENSCHIT00000016318 | eukaryotic translation initiation factor 2 subunit beta | K83I | 0.982* | 0.947 | possibly damaging |
|  |  | K205E | 0.907 | 0.528 | possibly damaging |
| ENSCHIT00000020934 | LY6/PLAUR domain containing 6B | A7T | 0.957* | 0 | benign |
|  |  | F16L | 0.911 | 0 | benign |
| ENSCHIT00000028741 | ATP binding cassette subfamily A member 12 | M570T | 0.904 | 0.74 | possibly damaging |
| ENSCHIT00000035903 | PATJ crumbs cell polarity complex component | V249I | 0.891 | 0.376 | benign |
|  |  | I1738F | 0.896 | 1 | probably damaging |
|  |  | I1739V | 0.864 | 0.012 | benign |
| ENSCHIT00000036547 | Rho GTPase activating protein 42 | I502L | 0.92 | 0.004 | benign |
|  |  | M770T | 0.986* | 0.001 | benign |
|  |  | W773R | 0.919 | 0.999 | probably damaging |
| ENSCHIT00000040177 | Achaete-scute family bHLH transcription factor 4 | L30S | 0.999** | 0.999 | probably damaging |
| ENSCHIT00000040379 | olfactory receptor 1P1 | A133T | 0.835 | 0.001 | benign |
|  |  | V135D | 0.928 | 0.795 | possibly damaging |
|  |  | H159C | 0.998** | 0.999 | probably damaging |
| ENSCHIT00000034768 | tripartite motif containing 16 | A155T | 0.694 | 0.95 | possibly damaging |
|  |  | D159L | 0.969* | 0.418 | benign |
|  |  | S515L | 0.872 | 0.151 | benign |
| ENSCHIT00000041152 | centrosomal protein 112 | K338G | 0.984* |  | unknown |

*Posterior probabilities were obtained from Bayes empirical Bayes; sites with posterior probabilities (95%) marked ** and * shows sites with high confidence that they are under strong signals of selections.

*Possible damaging and probably damaging are amino acid changes that are likely to change protein function and structure.
