# Supplementary material for: Genome-Wide Analysis of Nubian Ibex Reveals Candidate Positively Selected Genes That Contribute to Its Adaptation to the Desert Environment
Source: Animals (Basel). 2020 Nov 22;10(11):2181. doi: 10.3390/ani10112181 (PMC7700370; doi:10.3390/ani10112181)
Supplement: Supplementary file 1 [file animals-10-02181-s001.zip › Supplemental File S5. Gene ontology terms for positively selected genes.docx]

| **Gene ontology (GO) terms associated with positively selected genes in *C. nubiana*** | | | |
| --- | --- | --- | --- |
| **Ensembl id** | **Molecular functions** | **Cellular component** | **Biological process** |
| [ENSCHIT00000000612](http://www.ensembl.org/capra_hircus/Transcript/Summary?db=core;t=ENSCHIT00000000612) | GO:0005515~protein binding | GO:0005604~basement membrane,GO:0005615~extracellular space,GO:0031012~extracellular matrix,GO:0070062~extracellular exosome | GO:0001525~angiogenesis,GO:0030948~negative regulation of vascular endothelial growth factor receptor signaling pathway,GO:0090051~negative regulation of cell migration involved in sprouting angiogenesis |
| ENSCHIT00000003090 |  |  | GO:0001893~maternal placenta development,GO:0009790~embryo development |
| ENSCHIT00000004084 | GO:0005515~protein binding,GO:0008553~hydrogen-exporting ATPase activity, phosphorylative mechanism,GO:0046961~proton-transporting ATPase activity, rotational mechanism | GO:0001669~acrosomal vesicle,GO:0005829~cytosol,GO:0033178~proton-transporting two-sector ATPase complex, catalytic domain | GO:0008286~insulin receptor signaling pathway,GO:0015991~ATP hydrolysis coupled proton transport,GO:0016241~regulation of macroautophagy,GO:0033572~transferrin transport,GO:0034220~ion transmembrane transport,GO:0090383~phagosome acidification |
| ENSCHIT00000008957 | GO:0004252~serine-type endopeptidase activity | GO:0005783~endoplasmic reticulum | GO:0006508~proteolysis,GO:0043010~camera-type eye development |
| ENSCHIT00000010253 | GO:0005515~protein binding,GO:0043022~ribosome binding | GO:0005739~mitochondrion,GO:0005743~mitochondrial inner membrane,GO:0005759~mitochondrial matrix | GO:0007007~inner mitochondrial membrane organization,GO:0032979~protein insertion into mitochondrial membrane from inner side,GO:0036444~calcium ion transmembrane import into mitochondrion,GO:0051204~protein insertion into mitochondrial membrane,GO:0051560~mitochondrial calcium ion homeostasis,GO:0097033~mitochondrial respiratory chain complex III biogenesis,GO:0097034~mitochondrial respiratory chain complex IV biogenesis |
| ENSCHIT00000012782 | [GO:0004984](http://amigo.geneontology.org/amigo/term/GO:0004984)~olfactory receptor activity, [GO:0004930](http://amigo.geneontology.org/amigo/term/GO:0004930)~G protein-coupled receptor activity | [GO:0016021](http://amigo.geneontology.org/amigo/term/GO:0016021)~ integral component of membrane | [GO:0007186](http://amigo.geneontology.org/amigo/term/GO:0007186)~G protein-coupled receptor signaling pathway |
| ENSCHIT00000015750 | GO:0004497~monooxygenase activity,GO:0005506~iron ion binding,GO:0005515~protein binding,GO:0008116~prostaglandin-I synthase activity,GO:0016705~oxidoreductase activity, acting on paired donors, with incorporation or reduction of molecular oxygen,GO:0020037~heme binding | GO:0005615~extracellular space,GO:0005634~nucleus,GO:0005783~endoplasmic reticulum,GO:0005789~endoplasmic reticulum membrane,GO:0005901~caveola,GO:0016021~integral component of membrane | GO:0001516~prostaglandin biosynthetic process,GO:0006690~icosanoid metabolic process,GO:0006769~nicotinamide metabolic process,GO:0007566~embryo implantation,GO:0019371~cyclooxygenase pathway,GO:0032088~negative regulation of NF-kappaB transcription factor activity,GO:0035360~positive regulation of peroxisome proliferator activated receptor signaling pathway,GO:0045019~negative regulation of nitric oxide biosynthetic process,GO:0045766~positive regulation of angiogenesis,GO:0046697~decidualization,GO:0050728~negative regulation of inflammatory response,GO:0055114~oxidation-reduction process,GO:0071347~cellular response to interleukin-1,GO:0071354~cellular response to interleukin-6,GO:0071456~cellular response to hypoxia,GO:0097190~apoptotic signaling pathway,GO:1900119~positive regulation of execution phase of apoptosis |
| ENSCHIT00000016318 | GO:0003723~RNA binding,GO:0003743~translation initiation factor activity,GO:0005515~protein binding,GO:0008135~translation factor activity, RNA binding,GO:0044822~poly(A) RNA binding,GO:0046872~metal ion binding | GO:0005634~nucleus,GO:0005737~cytoplasm,GO:0005829~cytosol,GO:0005850~eukaryotic translation initiation factor 2 complex | GO:0001701~in utero embryonic development,GO:0002176~male germ cell proliferation,GO:0006413~translational initiation,GO:0008584~male gonad development,GO:0055085~transmembrane transport |
| ENSCHIT00000017349 | GO:0005524~ATP binding,GO:0008559~xenobiotic-transporting ATPase activity,GO:0015562~efflux transmembrane transporter activity,GO:0016887~ATPase activity,GO:0042626~ATPase activity, coupled to transmembrane movement of substances | GO:0005886~plasma membrane,GO:0005887~integral component of plasma membrane,GO:0016021~integral component of membrane | GO:0002481~antigen processing and presentation of exogenous protein antigen via MHC class Ib, TAP-dependent,GO:0002485~antigen processing and presentation of endogenous peptide antigen via MHC class I via ER pathway, TAP-dependent,GO:0002489~antigen processing and presentation of endogenous peptide antigen via MHC class Ib via ER pathway, TAP-dependent,GO:0002591~positive regulation of antigen processing and presentation of peptide antigen via MHC class I,GO:0006855~drug transmembrane transport,GO:0030154~cell differentiation,GO:0042391~regulation of membrane potential,GO:0042908~xenobiotic transport,GO:0048058~compound eye corneal lens development,GO:0055085~transmembrane transport |
| ENSCHIT00000018881 | GO:0003677~DNA binding,GO:0004842~ubiquitin-protein transferase activity | GO:0000151~ubiquitin ligase complex | GO:0006511~ubiquitin-dependent protein catabolic process,GO:0016567~protein ubiquitination |
| ENSCHIT00000020934 |  | GO:0005886~plasma membrane,GO:0031225~anchored component of membrane |  |
| ENSCHIT00000026283 | GO:0003700~transcription factor activity, sequence-specific DNA binding,GO:0043565~sequence-specific DNA binding,GO:0046872~metal ion binding | GO:0005634~nucleus,GO:0070062~extracellular exosome | GO:0006351~transcription, DNA-templated,GO:0006355~regulation of transcription, DNA-templated |
| ENSCHIT00000028741 | GO:0005102~receptor binding,GO:0005215~transporter activity,GO:0005319~lipid transporter activity,GO:0005515~protein binding,GO:0005524~ATP binding,GO:0016887~ATPase activity,GO:0034040~lipid-transporting ATPase activity,GO:0034191~apolipoprotein A-I receptor binding,GO:0042626~ATPase activity, coupled to transmembrane movement of substances | GO:0005737~cytoplasm,GO:0005743~mitochondrial inner membrane,GO:0005829~cytosol,GO:0005886~plasma membrane,GO:0016021~integral component of membrane,GO:0097209~epidermal lamellar body | GO:0006869~lipid transport,GO:0010875~positive regulation of cholesterol efflux,GO:0019725~cellular homeostasis,GO:0031424~keratinization,GO:0032940~secretion by cell,GO:0033700~phospholipid efflux,GO:0035627~ceramide transport,GO:0043129~surfactant homeostasis,GO:0045055~regulated exocytosis,GO:0048286~lung alveolus development,GO:0055085~transmembrane transport,GO:0055088~lipid homeostasis,GO:0061436~establishment of skin barrier,GO:0072659~protein localization to plasma membrane,GO:2000010~positive regulation of protein localization to cell surface |
| ENSCHIT00000028977 | GO:0000993~RNA polymerase II core binding,GO:0005515~protein binding | GO:0005654~nucleoplasm,GO:0005694~chromosome | GO:0006283~transcription-coupled nucleotide-excision repair,GO:0009411~response to UV,GO:0016567~protein ubiquitination |
| ENSCHIT00000030384 | GO:0004842~ubiquitin-protein transferase activity,GO:0005515~protein binding |  | GO:0006464~cellular protein modification process,GO:0006508~proteolysis,GO:0016567~protein ubiquitination, |
| ENSCHIT00000034768 |  |  | GO:0006355~regulation of transcription, DNA-templated,GO:0032526~response to retinoic acid,GO:0043966~histone H3 acetylation,GO:0043967~histone H4 acetylation,GO:0045618~positive regulation of keratinocyte differentiation,GO:0045893~positive regulation of transcription, DNA-templated,GO:0046683~response to organophosphorus,GO:0048386~positive regulation of retinoic acid receptor signaling pathway,GO:0050718~positive regulation of interleukin-1 beta secretion,GO:0060416~response to growth hormone |
| ENSCHIT00000035903 | GO:0005515~protein binding, | GO:0005886~plasma membrane,GO:0005923~bicellular tight junction,GO:0016324~apical plasma membrane,GO:0043234~protein complex,GO:0048471~perinuclear region of cytoplasm,GO:0070062~extracellular exosome | GO:0035556~intracellular signal transduction,GO:0070830~bicellular tight junction assembly, |
| ENSCHIT00000036547 | GO:0005096~GTPase activator activity |  | GO:0003085~negative regulation of systemic arterial blood pressure,GO:0007165~signal transduction,GO:0035024~negative regulation of Rho protein signal transduction,GO:0043547~positive regulation of GTPase activity,GO:1904694~negative regulation of vascular smooth muscle contraction |
| ENSCHIT00000040177 | GO:0000977~RNA polymerase II regulatory region sequence-specific DNA binding,GO:0003700~transcription factor activity, sequence-specific DNA binding,GO:0005515~protein binding,GO:0046983~protein dimerization activity | GO:0090575~RNA polymerase II transcription factor complex | GO:0006351~transcription, DNA-templated,GO:0006357~regulation of transcription from RNA polymerase II promoter,GO:0043588~skin development |
| ENSCHIT00000040379 | [GO:0004984](http://amigo.geneontology.org/amigo/term/GO:0004984)~olfactory receptor activity, [GO:0004930](http://amigo.geneontology.org/amigo/term/GO:0004930)~G protein-coupled receptor activity | [GO:0016021](http://amigo.geneontology.org/amigo/term/GO:0016021)~integral component of membrane | [GO:0007186](http://amigo.geneontology.org/amigo/term/GO:0007186)~G protein-coupled receptor signaling pathway |
| ENSCHIT00000041152 |  | GO:0005737~cytoplasm,GO:0005813~centrosome,GO:0005886~plasma membrane,GO:0060077~inhibitory synapse | GO:0097120~receptor localization to synapse, |
