# Supplementary material for: Genome-Wide Analysis of Nubian Ibex Reveals Candidate Positively Selected Genes That Contribute to Its Adaptation to the Desert Environment
Source: Animals (Basel). 2020 Nov 22;10(11):2181. doi: 10.3390/ani10112181 (PMC7700370; doi:10.3390/ani10112181)
Supplement: Supplementary file 1 [file animals-10-02181-s001.zip › Supplemental File S6. ASCL4 and UVSSA gene phylogenetic trees and sequence alignments used for positive selection analysis.pdf]

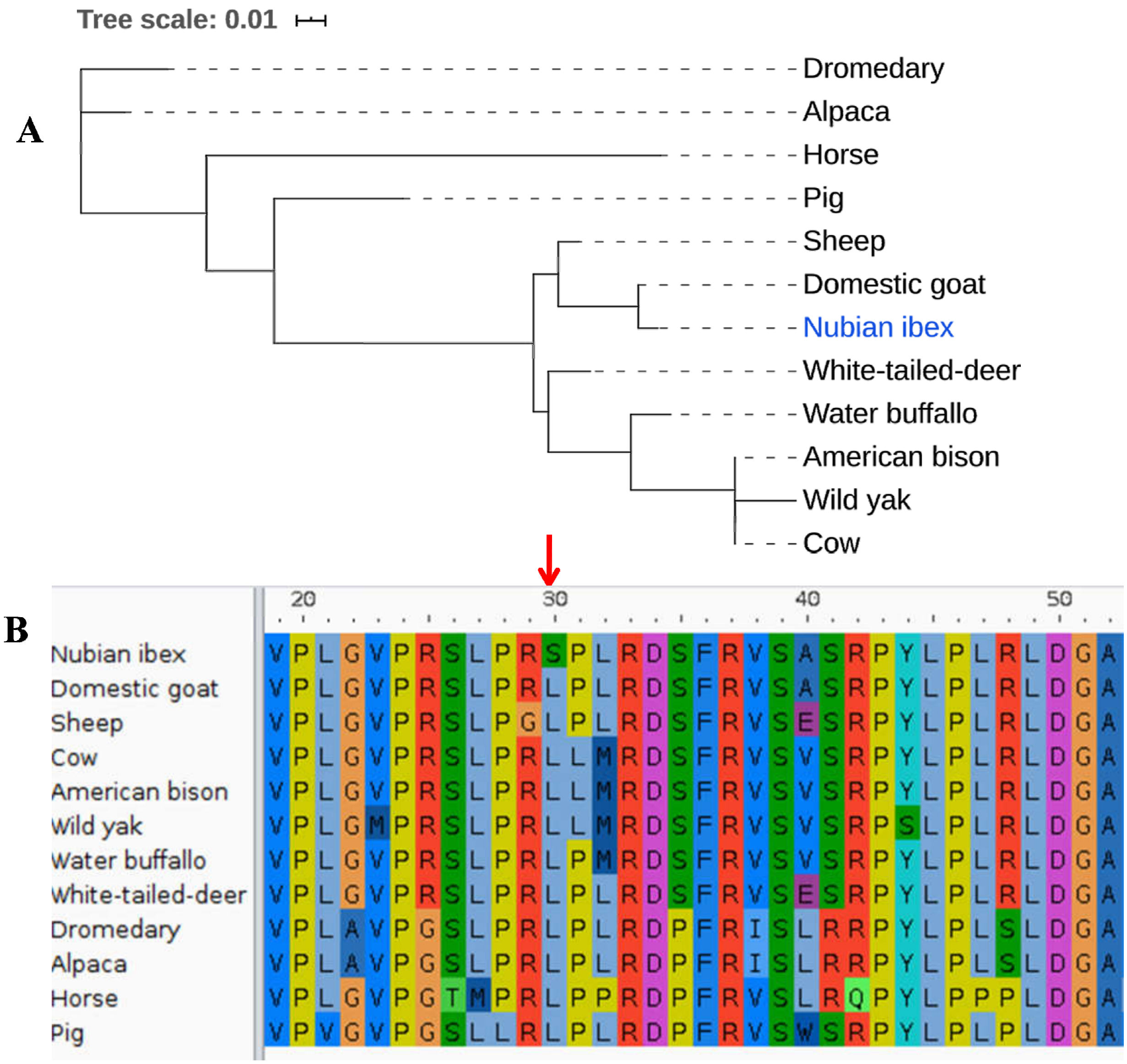


**Figure 1.** An illustration of the *ASCL4* gene phylogeny tree and alignment data used for dN/dS analysis. **A**. The maximum likelihood phylogenetic tree from *ASCL4* amino acid sequences of 12 species used for dN/dS analysis. **B**. The multiple sequence alignment of the 12 species used of dN/dS analysis showing amino acid site (L30S) under positive selection in *ASCL4* gene.

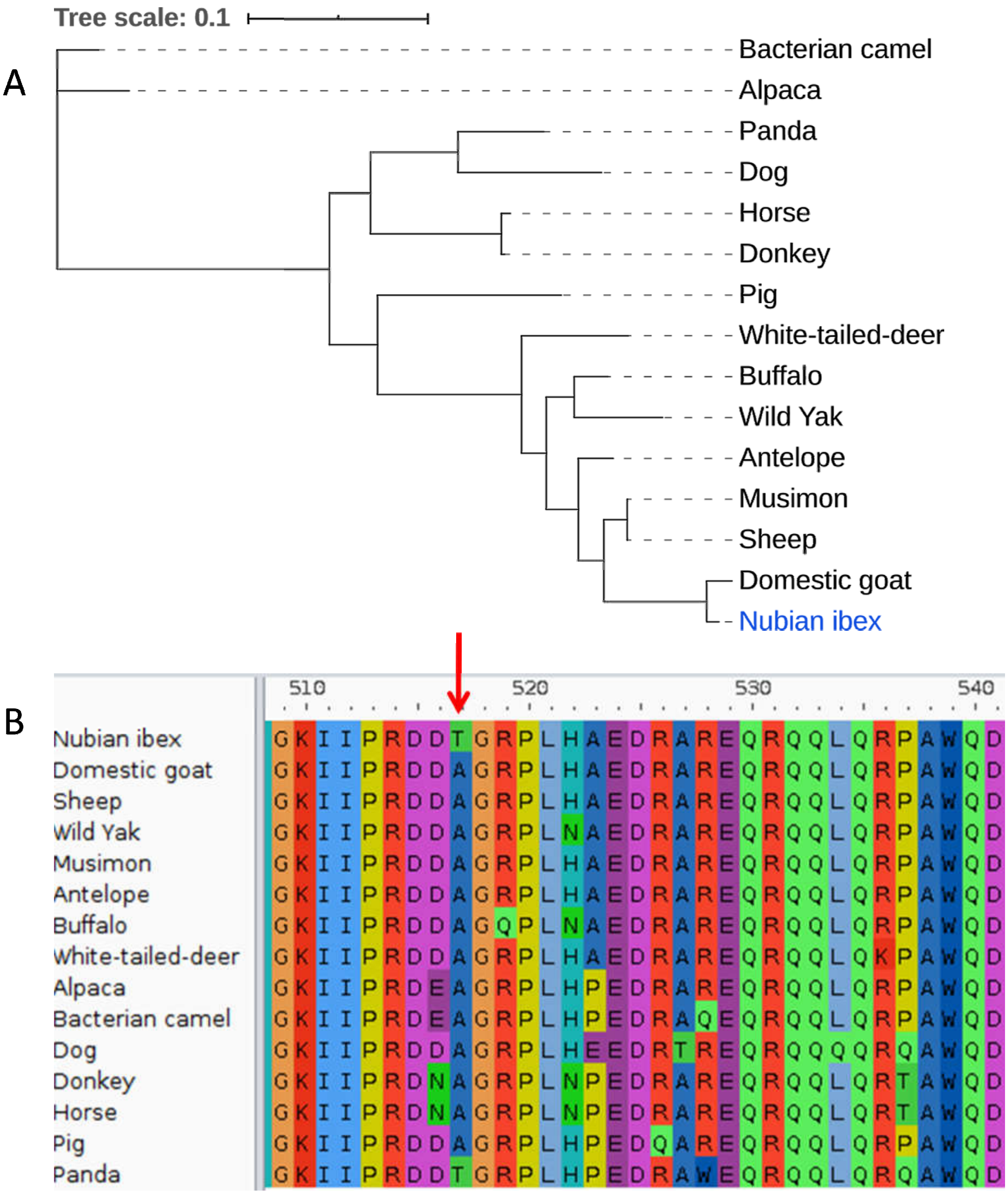


**Figure 2.** An illustration of the *UVSSA* gene phylogeny tree and alignment data used for dN/dS analysis. **A.** The maximum likelihood phylogenetic tree from *UVSSA* amino acid sequences of15 species used for dN/dS analysis. **B.** The multiple sequence alignment of the 15 species used of dN/dS analysis showing amino acid site (T517A) under positive selection in *UVSSA* gene.
